# Socio-demographic and geographic disparities of population-level food insecurity during the COVID-19 pandemic in Thailand

**DOI:** 10.3389/fpubh.2022.1071814

**Published:** 2023-01-13

**Authors:** Sirinya Phulkerd, Natjera Thongcharoenchupong, Aphichat Chamratrithirong, Rossarin Soottipong Gray, Umaporn Pattaravanich, Chantana Ungchusak, Pairoj Saonuam

**Affiliations:** ^1^Institute for Population and Social Research, Mahidol University, Nakhon Pathom, Thailand; ^2^Healthy Lifestyle Promotion Section of Thai Health Promotion Foundation, Bangkok, Thailand

**Keywords:** food insecurity, socio-demographic characteristics, geographical areas, COVID-19, Thailand

## Abstract

**Introduction:**

This study investigated the prevalence of food insecurity, and the association between socio-demographic and geographic factors and food insecurity in Thailand during the COVID-19 pandemic.

**Methods:**

The study extracted data on 5,066 persons age 15 years or older from a nationally-representative sample survey of Thai households, conducted during June-December 2021. The respondents were asked about food insecurity, socio-demographic characteristics, debt, and role of the primary household food provider. Binary logistic regression analysis was used to investigate the association between the variables and food insecurity.

**Results:**

Of the total sample, 28.6% had food insecurity. The highest probability of having food insecurity (*p* < 0.001) was observed in persons age 15–29 years, with no formal education, and in the lowest quartile of income. The highest probability of having food insecurity was found among respondents residing in the northeast, which is the poorest and with the least development status among geographic regions in Thailand. Respondents who reported having onerous personal debt and being the main household food provider were 1.4 and 2.3 times as likely to have food insecurity as those with no debt and not being the main food provider, respectively (*p* < 0.001).

**Conclusion:**

This suggests that government attention is required in developing policies and strategies to improve food security through addressing the socio-economic determinants, and buffer the negative impact of a national crisis on diets. Investment to improve household income and raise the educational profile of the population is needed. Addressing the regional disparities in food security requires area-specific measures which target the most vulnerable population groups.

## 1. Introduction

In 2022, the COVID-19 pandemic has continued to wreak havoc in nearly every country in the world, with more than 520 million confirmed cases and more than six million deaths reported as of May 23, 2022 (1). Among the disastrous health and economic impacts of COVID-19, the pandemic has had profound effects on food security and nutrition of the global population (2). Food security and adequate nutrition were already fragile in many countries before 2020. However, the pandemic has made the situation drastically worse. In 2020, more than 700 million people around the world faced hunger, which was 118 million more people than in 2019. A year later, more than two billion people did not have access to adequate food, which was an increase of 320 million from 2019. Combined with natural disasters, human conflict, and a collapse in global demand for internationally-produced agro-food products, the COVID-19 pandemic has made food insecurity immeasurably worse (2).

Failure to improve food security can lead to many adverse consequences. Mounting evidence points to an association of food insecurity with poverty, poor health, and stunted human development. For example, among Mexican families, being above the poverty line was associated with lower food insecurity (3). Another study found that food insecurity adversely affected growth, cognitive, and behavioral potential of America's poor and near-poor children (4). Previous research also found an association between food insecurity and health consequences in specific population groups, e.g., food insecurity was associated with decreased nutrient intake in children and adults (5, 6); mental health problems and depression (7, 8), diabetes (9), hypertension in adults (10); and depression and anxiety in mothers (11). Food insecurity also affected healthcare utilization and expenditure (12). Furthermore, healthcare utilization and expenditures were likely to increase when household food insecurity increased.

Determinants of food insecurity and the mechanisms through which it impacts on vulnerable households can differ according to physical, social, and economic contexts in which people are living. This includes demographic and geographic contexts which interact in ways already evident elsewhere. In India, a higher percentage of older adults who were hungry did not eat enough food because there were shortages in the household, and this phenomenon was more prevalent in rural than in urban areas (13). In Mexico, vulnerable population (e.g., fishing families) were associated with severity of food insecurity (3). In Iran, a lower employment status and educational level of the household head were significant predictors of food insecurity (14). There is also evidence showing that personal savings, household income, employment status of head of household had a significant association with food insecurity during the COVID-19 pandemic in Iran (15). Similarly, another Iranian study reported arduous economic conditions as important contributors to household food insecurity during the initial COVID-19 lockdown (16). In Canada household food insecurity and the severity of the experience were strongly associated with province or territory of residence (17).

Thailand is among many countries in Southeast Asia which have experienced multiple waves of spread of COVID-19 (18). The 1st wave of Thailand's pandemic started in March 2020, second wave in December 2020, third wave in April 2021, forth wave in June 2021 and fifth wave in January 2022. The Thai government declared and enforced a State of Emergency Decree on 26 March 2020 and curfews between 22:00 and 04:00 (19). The government also announced a number of public health and social measures to control or mitigate the COVID-19, such as lockdowns of various duration and scope, closing of entertainment establishments and sit-down dining, and closures of schools to in-person classroom learning. Access to public spaces was restricted.

There were many socio-economic repercussions of these harsh restrictions, and these measures both directly and indirectly disrupted the food supply chain and consumption of essential nutrients (2). In particular many people in urban areas suddenly had limited access to fresh and nutritious foods. In the year prior to the onset of the Thai pandemic, 39% of Thai households with children under 5 years old had some worries that they would not have enough food to properly feed their child(ren), 25% were unable to eat healthy and nutritious food, and 8% had to skip a meal (20). Among all sample households, 3% were already identified as moderate or severe food-insecure. The poorest Thai households were most likely to suffer from food insecurity, and urban households experienced greater severity of food insecurity than households in rural areas. However, that survey was only conducted with families with a child under 5 years old, and the subsample was unlikely to be representative of the situation of all households nationwide.

Conditions that give rise to population-level food insecurity in Thailand remain poorly understood. As yet, no studies had investigated the prevalence of food insecurity in the Thai population, especially during the COVID-19 pandemic, nor had any studies identified the determinants that affect vulnerability to food insecurity. Unfortunately, combatting food insecurity has not been a priority for public policy intervention in Thailand.

Therefore, the objective of this study was to investigate the prevalence of food insecurity in the Thai population, and socio-demographic and geographic factors associated with food insecurity during the COVID-19 pandemic. Findings of this study should strengthen understanding of how to ensure adequate availability, access, and consumption of nutritious food. The findings at the disaggregated geographical level will allow policymakers and programme planners to visualize which regions or area of residence are most in need of policies and strategies to guarantee the right to secure food. The findings should help guide the way toward accelerated achievement of Sustainable Development Goal 2 (SDG2) — in particular SDG Target 2.1 (“*Ensuring access to safe, nutritious, and sufficient food for all people all year round”*) and SDG Target 2.2 (“*Eradicating all forms of malnutrition”)* (21).

## 2. Materials and methods

### 2.1. Study design and population

This study used data that were collected in 2021 (during the 3rd and 4th waves of the COVID-19 pandemic in Thailand) from a nationally-representative household survey of the Thai population, namely, “*A Cross-sectional Study on Fruit and Vegetable Eating Behaviors”* (22). For the purpose of this study, a subsample of the dataset was selected for those age 15 years or older.

The survey sampling was conducted by the National Statistical Office (NSO) which applied a multi-stage sampling design. The sampling frame was a hierarchical structure in which households are nested within sampled region, province, district, and enumeration area, respectively. Within each sampled district from nine selected provinces, enumeration areas were randomly chosen from a nationally-representative sampling frame used in the national Population and Housing Census. Twenty households were selected from each enumeration area.

### 2.2. Data collection

Data were collected during June-December 2021 using Qualtrics offline survey application. The survey questionnaire includes items on socio-demographic characteristics, health status, personal debt, and food insecurity. Responses were recorded in the field on a digital device, and then uploaded to Qualtrics as soon as an Internet connection was available. The data collection was carried out by a trained research team.

The questions about food insecurity used the Food Insecurity Experience Scale (FIES), which is experience-based measures of household or individual food security, developed by the Food and Agriculture Organization (FAO) (23). A Thai version of FIES was pretested to evaluate reliability and internal consistency of the questions before actual data collection.

The survey included households in the four geographic regions of Thailand (central, north, northeast, and south) and Bangkok, and included both urban and rural areas in each region. A total sample of 5,066 subjects were included for analysis.

The research team contacted local coordinators such as community leaders and village health volunteers in each study area as liaisons to help the interviewers approach the sampled households. All subjects received a description of the survey and purpose of the study, and written informed consent was obtained prior to the interview.

### 2.3. Outcome variables

Food insecurity was measured through eight questions developed by FAO (Table 1). Each respondent was asked about his or her food-related behavior, and experience associated with increased difficulty in accessing food due to resource constraints. Each question refers to a different experience and represents a different level of severity of food insecurity.

**Table 1:** FIES questions for measuring severity of food insecurity.

**Question**	**Standard label**	**Severity scale**
[Opening question] *Now I would like to ask you some questions about food. During the last 12 months, was there a time when:*		
Q1 You were worried you would not have enough food to eat?	WORRIED	**Mild food insecurity** (Worrying about running out of food) 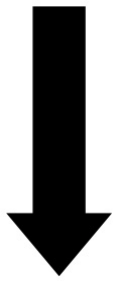 **Severe food insecurity** (Experiencing hunger)
Q2 You were unable to eat healthy and nutritious food?	HEALTHY	
Q3 You ate only a few kinds of foods?	FEWFOODS	
Q4 You had to skip a meal?	SKIPPED	
Q5 You ate less than you thought you should?	ATELESS	
Q6 Your household ran out of food?	RANOUT	
Q7 You were hungry but did not eat?	HUNGRY	
Q8 You went without eating for a whole day?	WHOLEDAY	

The individual respondent was asked to give a “*yes/no*” response to each question. The questions were analyzed together as a scale (i.e., not as separate questions) to assess a range of severity of food insecurity. A respondent who answered “*yes*” continued to answer the follow-up questions; a respondent answering “*no*” did not need to proceed further with follow-up questions. Response to each question was assigned a potential score of one point. The respondent's score was the sum of the eight FIES questions, with a potential total ranging from zero to eight. The more questions which the respondent answered “*yes*” to, the more severe their food insecurity (i.e., with a higher score). A response of “no” since the first question was coded as “*food secure*.” This classification is based on previous literature using FIES (20, 24–27).

Prior to data collection the statistical analysis to assess the internal consistency (reliability) for the set of Thai FIES questions was performed using Cronbach's alpha. Previous literature indicates that an acceptable range of values of alpha is from 0.70 to 0.95 (28, 29). The alpha value of the Thai FIES in this study was 0.89, indicating good reliability.

### 2.4. Independent variables

#### 2.4.1. Socio-demographic factors

Socio-demographic variables included the following:

Sex was coded as male and female;

Age was coded as early-working age (15–29 years), middle-working age (30–44 years), late-working age (45–59 years) and older person (60 years or older).

Marital status was coded as married, single, or widowed/divorced/separated.

Educational attainment was coded as currently studying, no formal education/primary not completed, primary school, secondary school, and bachelor's degree or higher.

Income quartile (Q) was based on the median income of the respondents, with Q1 being the lowest income and Q4 being the highest. This variable was coded as Q1 (0–4,999 baht), Q2 (5,000–8,999 baht), Q3 (9,000–14,999 baht), and Q4 (15,000–300,000 baht).

Current occupation was coded as unemployed (people who are not in the labor force, jobless, actively seeking work, available to take a job), government employee, company-hired worker, business owner, wage laborer, and farmer.

Personal debt was measured based on data of the NSO Household Socio-economic Survey (2013, 2015, 2017, 2019) and estimation of Bank of Thailand (30). This variable reflects the impact of debt burden on household consumption during the COVID-19pandemic. Debt was coded as “*yes*” (in debt), and “*no*” (without debt).

#### 2.4.2. Geographic factors

Place of residence was coded as either rural or urban. Region of residence was coded as either central, north, northeast, south, or Bangkok.

#### 2.4.3. Health and food-related factors

Health status and food provision were included in the analysis. The respondents were asked to self-assess their health status. Respondent's health status was coded as “*yes*” (currently have a chronic disease) and “*no*” (no chronic disease). Respondents were also asked whether they are the main food provider in the household, with response coded as “*yes*” or “*no*.”

### 2.5. Statistical analysis

This study used descriptive statistics (i.e., frequencies and percentages) in describing prevalence of food insecurity for the set of FIES questions, presence of food insecurity, socio-demographic and geographic factors, and health- and food-related behaviors. To test for statistically-significant associations between food insecurity and respondent characteristics, binary logistic regression analysis was employed, regressing the bi-level individual food insecurity (i.e., having/not having food insecurity) on the socio-demographic and geographic characteristics of the respondents.

Odds ratios (ORs) were calculated, and are presented with a 95% confidence interval (CI). All of the variables were calculated to yield an adjusted odds ratio of food insecurity. The relationship was considered statistically significant if the *p* value was 0.05 or less (2-tailed). All analyses were conducted using SPSS version 22.

## 3. Results

Table 2 presents prevalence of food insecurity by socio-demographic and geographic characteristics of the sample. Of the total 5,066 respondents, more than one-fourth (28.6%) reported having food insecurity. Groups of the respondents with the highest prevalence of food insecurity were women (33.5%), age 45–59 years (30.5%), married/cohabiting (30.8%), with no formal education (39.9%), with Q1 income (33.7%), and unemployed or wage laborer (35.3%).

**Table 2:** Characteristics of the sample by prevalence of food security/insecurity (*N* = 5,066).

**Variables**	***N***	**% of total**	**Food secure (score = 0)**	**Food insecure** **(score = 1–8)**	**Chi-square (*p*-Value)**
**Total**	**5,066**	**100.0**	**71.4**	**28.6**	
**Sex**					65.04^***^
Male	2,439	48.1	76.7	23.3	
Female	2,627	51.9	66.5	33.5	
**Age group**					10.29^*^
15–29 years	1,053	20.8	75.0	25.0	
30–44 years	983	19.4	71.7	28.3	
45–59 years	1,848	37.5	69.5	30.5	
60 years or over	1,182	23.3	70.9	29.1	
**Marital status**					38.55^***^
Single	1,157	22.8	78.7	21.3	
Married	3,214	63.5	69.2	30.8	
Widowed/divorced/separated	694	13.7	69.6	30.4	
**Place of residence**					26.45^***^
Urban	2,318	45.8	75.0	25.0	
Rural	2,748	54.2	68.4	31.6	
**Region of residence**					428.92^***^
Bangkok	513	10.1	95.7	4.3	
Central	917	18.1	82.1	17.9	
North	1,180	23.3	63.8	36.2	
Northeast	1,119	22.1	53.4	46.6	
South	1,337	26.4	76.5	23.5	
**Educational attainment**					97.63^***^
Currently studying	550	10.9	78.9	21.1	
No formal education	301	5.9	60.1	39.9	
Primary school	2,019	39.9	66.5	33.5	
Secondary school	1,732	34.2	73.2	26.8	
Bachelor's or higher degree	463	9.1	84.2	15.8	
**Income quartile**					148.83^***^
Q1 (0-4,999)	2,515	49.6	66.3	33.7	
Q2 (5,000-8,999)	917	18.2	67.8	32.2	
Q3 (9,000-14,999)	792	15.6	74.6	25.4	
Q4 (15,000-300,000)	842	16.6	87.5	12.5	
**Occupation**					85.92^***^
Unemployed	1,927	38.0	64.7	35.3	
Government employee	189	3.7	85.7	14.3	
Company-hired worker	350	6.9	85.4	14.6	
Business owner	900	17.9	74.6	25.4	
Wage laborer	761	15.0	64.7	35.3	
Farmer	939	18.5	66.2	33.8	
**Presence of a chronic disease**					7.981^**^
Yes	1,896	37.4	69.1	30.9	
No	3,170	62.6	72.8	27.2	
**Personal debt**					58.46^***^
Yes	2,079	41.0	65.6	34.4	
No	2,987	59.0	75.5	24.5	
**Main food provider in the household**					167.49^***^
Yes	2,482	49.0	63.0	37.0	
No	2,584	51.0	79.5	20.5	

* Sig. ≤ 0.05,

** Sig ≤ 0.01,

*** and Sig ≤ 0.001.

People who lived in a rural area (31.6%) and in the northeast region (46.6%) had the highest prevalence of food insecurity. About one-third of the respondents with a chronic disease (30.9%) or onerous debt (34.4%) were experiencing food insecurity. More of the respondents who reported being the primary food provider for the household suffered from food insecurity, compared to those who were not the main food provider. Statistically-significant differences were found in relation to all variables.

### 3.1. Food insecurity by FIES questions

**Figure 1:**
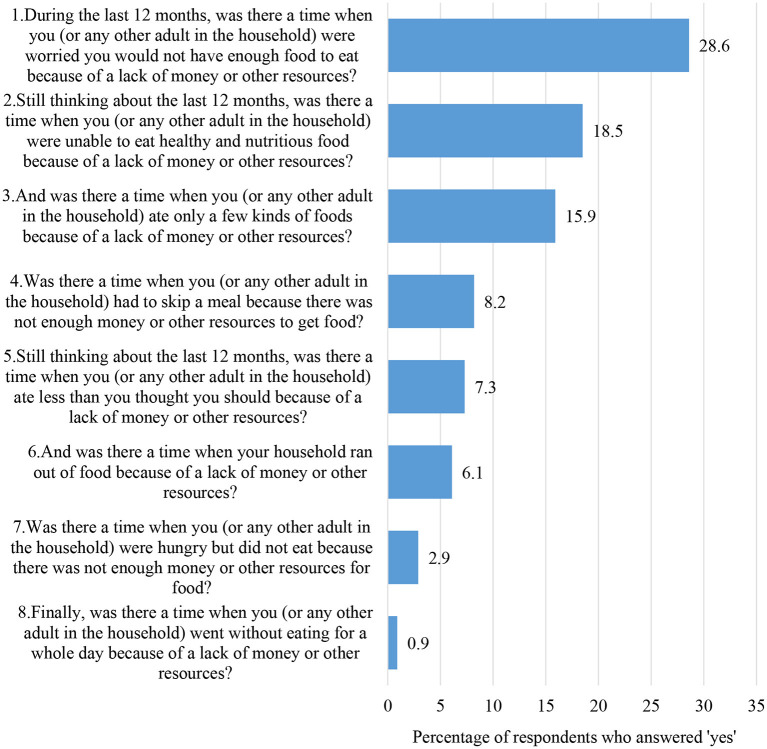
Respondents who answered “*yes*” by each of eight FIES questions (%).

By stratifying level of food insecurity, 28.6% of the respondents felt worried about not having an adequate amount food to eat; 18.5% could not eat enough healthy and nutritious food; 15.9% had limitations in eating several kinds of foods; 8.2% had to skip a meal; 7.3% ate less food than they should; 6.1% ran out of food in the household; 2.9% felt hungry but did not have food to eat; and 0.9% were deprived of food for a whole day (Figure 1).

### 3.2. Socio-demographic and geographic disparities in food insecurity

Table 3 shows results from the binary logistic regression analysis that tested the association between various socio-demographic and geographic factors and food insecurity. The analysis found that socio-demographic factors (i.e., education, occupation, and income) were significantly associated with food insecurity. Respondents who had no formal, primary school, or secondary school education were 2.7, 2.0, and 1.4 times as likely to have food insecurity as those with a bachelor's or higher degree (95% CI 1.787–4.084, 1.435–2.779 and 1.020–1.889, respectively). The *lowest* probability of having food insecurity was observed among people who were unemployed (95% CI 0.510–0.806). The *highest* probability of having food insecurity was observed among people with Q1 income (95% CI 2.905–5.410). The lowest-income group was 4.0 times as likely to have food insecurity compared to those in the highest income group, followed by Q2 (2.3 times) and Q3 (2.0 times) groups, respectively.

**Table 3:** Sample characteristics and food insecurity (*N* = 5,066).

**Variables**	**Exp (B)**	**Adjusted OR (95% CI)**	
		**Lower**	**Upper**
**Sex (Reference group** = **Male)**			
Female	1.030	0.880	1.206
**Age group (Reference group** = **60**+ **years)**			
15–29 yrs.	2.040^***^	1.485	2.804
30–44 yrs.	1.660^***^	1.279	2.156
45–59 yrs.	1.162	0.956	1.413
**Marital status (Reference group** = **Widowed/divorced/separated)**			
Single	1.248	0.979	1.591
Married	1.135	0.840	1.533
**Place of residence (Reference group** = **Urban)**			
Rural	1.042	0.906	1.199
**Region of residence (Reference group** = **South)**			
Bangkok	0.175^***^	0.110	0.279
Central	0.690^**^	0.552	0.862
North	1.724^***^	1.433	2.074
Northeast	2.645^***^	2.197	3.183
**Educational attainment**			
**(Reference group** = **Bachelor's or higher degree)**			
Currently studying	1.164	0.769	1.763
No formal education	2.702^***^	1.787	4.084
Primary school	1.997^***^	1.435	2.779
Secondary school	1.388^*^	1.020	1.889
**Occupation (Reference group** = **Wage laborer)**			
Unemployed	0.546^***^	0.420	0.709
Government employee	0.561^*^	0.345	0.911
Company-hired worker	1.085	0.735	1.601
Own business	0.898	0.705	1.144
Farmer	0.641^***^	0.510	0.806
**Income quartile (Reference group** = **Q4)**			
Q1 (0–4,999 baht)	3.965^***^	2.905	5.410
Q2 (5,000–8,999 baht)	2.331^***^	1.758	3.092
Q3 (9,000–14,999 baht)	1.972^***^	1.490	2.610
**Indebted (Reference group** = **No)**			
Yes	1.365^***^	1.181	1.579
**Chronic disease (Reference group** = **No)**			
Yes	1.060	0.909	1.236
**Main food provider in the household (Reference group** = **No)**			
Yes	2.267^***^	1.934	2.658
**Cox and snell R2**	**0.155**		

* Sig. ≤ 0.05,

** Sig ≤ 0.01,

*** and Sig ≤ 0.001.

By geographic region, the *highest* probability of having food insecurity was found among respondents residing in the northeast region (95% CI 2.197–3.183). Those respondents were 2.7 times as likely to have food insecurity as those residing in the south region. On the other hand, the *lowest* probability of having food insecurity was found among respondents residing in Bangkok (95% CI 0.110–0.279), with 82.5% as less likely to have food insecurity as those in the south region. There was no significant association of place of residence (urban/rural) with food insecurity.

Other associated factors were having onerous, personal debt and being the main food provider of the household. Respondents who reported having debt were 1.4 times as likely to have food insecurity as those with no debt (95% CI 1.181–1.579). Respondents who were the main food provider in the household were 2.3 times as likely to have food insecurity as those who were not the principal food provider (95% CI 1.934–2.658).

## 4. Discussion

Drawing on the data from a nationally-representative sample of the Thai population, this study investigated prevalence of food insecurity in Thailand during the COVID-19 pandemic, and its association with socio-demographic and geographic disparities. To our knowledge, this is the first investigation using a national household survey to measure food insecurity and associated factors at the population level. This study builds upon a previous study by Jankhotkaew et al. (20) with a representative sample of the Thai population, the response to the current health emergency, and an emphasis on the role of socio-demographic characteristics and geography of residence in relation to food insecurity.

In 2021, the prevalence of food insecurity in the Thai population was 28.6% (meaning that they at least worried about not having enough food to eat). This prevalence rate is higher than in many countries using the same measurement tool, e.g., Jordan (24), India (31), and Bangladesh (26). The differing results can be due to variations in demography, culture, and the survey methods used. The present study also found that food insecurity differs by certain socio-demographic characteristics, geographic region, and other factors, in particular, personal indebtedness and being the main food provider in the household.

n terms of socio-demographic disparities, this study found that the younger the population, the higher the food insecurity. That finding is consistent with previous research in young adults (32, 33). This finding could be due to anxiety about not being able to access certain foods, which they could do so easily before the pandemic. In particular, teenagers tend to prefer western “fast food” to traditional food, and the fast food outlets are concentrated in shopping malls, most of which were closed during various waves of the pandemic, or limited to take-out only (34). Even before the advent of COVID-19, there was ample evidence that Thai youth were developing unhealthy eating behaviors, such as overconsumption of cheaper, calorie-dense, nutrient-poor foods, and sugar-sweetened soft drinks (35, 36). Despite Thailand having some of the best fruit and vegetable options of countries around the world, Thai youth are eating less of these nutritious fruits and vegetables (37). During the Thai COVID-19 pandemic various containment measures (e.g., lockdowns) were implemented, and that severely limited consumer access to services, including retail food services and fresh markets.

During COVID-19, millions of Thais suddenly became unemployed or underemployed due to business closures and production slow-downs. However, contrary to expectation, the unemployed portion of the sample in this study had the *lowest* probability of being food insecure. This counter-intuitive finding may be explained by the fact that people who lost jobs returned to the home community, in which the extended family could pool resources and stretch budgets to ensure that everyone in the household got fed. By contrast, employed people were more likely to be in urban areas, and cities and towns were more adversely affected by lockdowns and closures than the rural families, who know how to “*live off the land*.” Other research also found an association between being employed during the COVID-19 pandemic and food insecurity (26, 38). During the pandemic, many workers in the service sector could not “work from home” and are generally paid only subsistence wages (39). Thus, they were much more vulnerable to the hardships of COVID-19-related lay-offs or reduction in work hours since they probably had little savings to rely on. Consequently, those who still managed to remain employed were probably living on the margins, and would have had difficulty making ends meet and having enough income for proper nutrition and food sufficiency (40, 41). This study suggests that government attention is required in developing social support policies and systems to improve population food security, health, and wellbeing through addressing the socioeconomic determinants and buffer the impact of a national crisis, especially for vulnerable groups. This action should be done with community and civil organization engagement. This will open up opportunities for people to cope with calamity, improve their economic status, and ultimately improve their food security.

Thai people in the northeastern region had the highest probability of having food insecurity, and those in Bangkok were least likely to having food insecurity. In Thailand, there are significant differences in socio-economic development among the four geographic regions. The north, northeast, and south lag behind Bangkok and the central region in terms of economic growth and social development (42). The northeast region is especially being left behind, with the lowest gross regional product per capita in 2020, accounting for 86,233 baht per annum, whereas Bangkok and vicinity recorded 436,255 baht per annum per capita (43). In addition, the northeast is a predominantly agriculture-based region, and is largely covered by sandy soil, which is less fertile for cultivation and more prone to drought and flooding (44). Despite overall low food insecurity in Bangkok, people living in slums-more than half, were still hungry but did not eat due to financial constraints (45). Accordingly, the sociodemographic and geographic disparities increase the challenge to equalize food security. That said, modern innovations and advanced technologies in agro-food production and processing are available and needed, but applying those assets also requires a business environment that is conducive to investment in promoting access to healthy food in long-term.

Other factors associated with food insecurity were personal debt and being the main provider of food for the household. The impact of indebtedness on food insecurity has been proven, especially during a pandemic. Previous research found that many people took on extra debt during the COVID-19 (46). Spending on nutritious food is one of the first personal budget items to be cut back on by a debtor (38, 46, 47). The link between being the main food provider of the household and food insecurity may be explained by the added hardship of the person who has to provide sustenance for all other members of the household, especially in large extended families. As noted, the COVID-19 pandemic strained food supply, and the reduced disposable income hampered the household's ability to access and provide a nutritious and balanced diet every day (48, 49). It was also reported elsewhere that people resorted to ultra-processed food and beverage products during times of scarcity, and those foods are often high in fat, sugar, and sodium — though they may have a longer shelf life and create a sensation of being full. Fresh fruits, vegetables, and meats were less available during periods of lockdown, and the more nutritious foods usually require refrigeration to stay fresh (50, 51).

The stress of the main household food provider could aggravate poor eating behaviors, and that may lead to higher risk of food insecurity and worse healthy status later in life. The findings suggest that government action, such as investment in community-based food banks, may be needed to help support nutritious diets for people in need. Improvements in the food supply chain are needed, for example, by shortening the farm-to-pantry process, and promoting consumption of locally-grown food, such as kitchen gardens and local food cooperatives (52, 53).

There are some limitations of this study. First, the study collected data *via* a self-report questionnaire, and response may be subject to recall bias. Second, the data were collected at a single point of time and, thus, it is not possible to draw firm conclusions about the significance and direction of the impact of the COVID-19 on food insecurity. Future research should use a prospective or longitudinal approach to better understand relationships between factors and food insecurity. That would also help forecast longer-term trends in food insecurity in Thailand. Third, other unmeasured factors may be more important determinants of food insecurity (such as food culture and norms specific to each geographic region, or other socio-economic factors). Future studies should expand the number of independent variables in the data collection.

## 5. Conclusions

This study found that food insecurity is still a significant threat in Thailand, with over one-fourth of the population having experienced food insecurity during the COVID-19 pandemic. Food insecurity differs according to various socio-demographic and geographic factors. The findings indicate that food insecurity might be positively affected by age, education, income, and region of residence. The findings suggest that more government attention should be paid to policies and strategies to improve food security through addressing the socio-economic determinants and ways to buffer the impact of a crisis on unhealthy food consumption. Long-term investment in improving incomes and raising the educational profile of the population, especially marginalized groups, are needed. National policy and programs for food security should give priority to those parts of the country which lag behind the other regions. The requisite innovations and advanced technologies in agro-food production and processing already exist. These need to be applied in an enabling and sustainable environment that is conducive to new businesses and investment in healthy food in the long-term.

## Data availability statement

The raw data supporting the conclusions of this article will be made available by the authors, upon reasonable request.

## Ethics statement

The studies involving human participants were reviewed and approved by the Institutional Review Board of the Institute for Population and Social Research of Mahidol University. Written informed consent to participate in this study was provided by the participants' legal guardian/next of kin.

## Author contributions

SP: conceptualization, formal analysis, funding acquisition, investigation, methodology, project administration, visualization, writing—original draft preparation, and writing—review and editing. NT: formal analysis, investigation, methodology, and writing—review and editing. AC, RG, and UP: conceptualization, methodology, and writing—review and editing. CU and PS: writing–review and editing. All authors contributed to the article and approved the submitted version.
